# Poliomyelitis in Pakistan: Challenges to polio eradication and future prospects

**DOI:** 10.1016/j.amsu.2022.104274

**Published:** 2022-07-31

**Authors:** Haroon Shabbir, Sajeel Saeed, Muhammad Farhan, Khawar Abbas, Mohammad Ebad ur Rehman, Fahad Gul, Jawad Basit

**Affiliations:** a Department of Medicine, Rawalpindi Medical University, Rawalpindi, Pakistan; b Department of Surgery, Rawalpindi Medical University, Rawalpindi, Pakistan

**Keywords:** Poliomyelitis, Polio virus (PV), Post-polio syndrome (PPS), Inactivated polio vaccine (IPV), Oral polio vaccine (OPV), Vaccine associated paralytic poliovirus (VAPP), Paralytic poliovirus, Wild polio virus (WPV)

## Abstract

Poliomyelitis is a viral disease that causes acute paralysis, muscle weakness and autonomic dysfunction. It primarily affects children under the age of five. It is mainly transmitted via the feco-oral route, through contaminated water. As of the year 2022, Pakistan remains one of the two countries where polio is still endemic, the other being Afghanistan. Numerous myths and misconceptions regarding the polio vaccine, lack of awareness and proper governance, terrorism and difficult access to remote areas due to poor infrastructure are just some of the reasons why polio remains endemic in Pakistan to this day. Therefore, the government should take measures to ensure the safety and wellbeing of health care workers, as well as spread awareness regarding the importance of polio vaccines, while addressing the myths and misconception regarding said vaccines.

Dear Editor,

Poliomyelitis is an acute paralytic disease that primarily affects children under the age of five. It is caused by a single-stranded positive-sense RNA virus i.e., Polio Virus (PV), which is found in three serotypes (type 1, 2, and 3). Owing to the Global Polio Eradication initiative by the World Health Organization, since 1988, there has been a 99.9% decline in polio cases across the globe [1]. Around three billion children have been vaccinated by this program over the past 33 years. As of 2022, Pakistan remains one of the two countries where polio is still endemic [1]. Fig. 1 has shown the number of cases from 2015 till now in Pakistan year-wise [2].

**Fig. 1:**
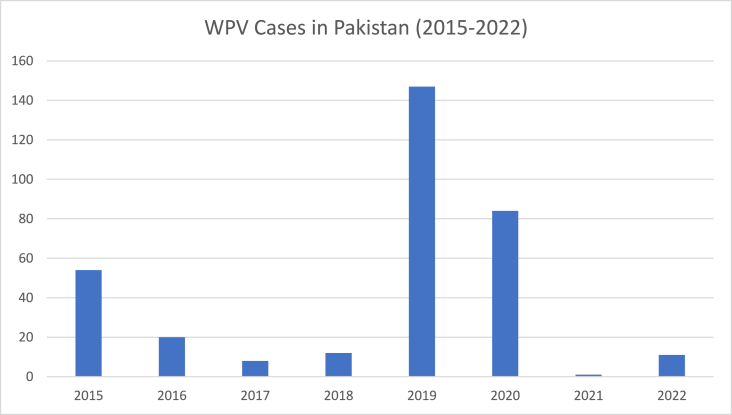
WPV cases in Pakistan.

Poliovirus is a member of the *Picornaviridae* family and species Enterovirus C [3]. Of its three serotypes, types 2 and 3 are considered eradicated as of 2015 [4]. Poliovirus is the primary causative agent of both acute polio and Post-Polio Syndrome (PPS). Poliomyelitis prognosis is characterized by three distinct phases i.e., acute, recovery, and the residual-paralysis phase. Patients in the acute phase present with pyrexia, paraparesis, muscular weakness, and autonomic dysfunction [4]. The number of muscle fibers innervated by a single motor neuron i.e., motor unit, increases during the recovery phase. The patient is left with imbalanced muscle power, poor posture, and residual paralysis in the last phase. Of the total cases, around five percent develop paralysis. The remaining infections are non-paralytic. Regarding Post-Polio Syndrome, it is a progressive disorder characterized by muscular weakness, joint pain, and tiredness, occurring in people many years after they have had polio.

Virus isolation in culture is the most sensitive method to diagnose polio. A sample for said culture may be obtained from the throat, stool, or Cerebrospinal Fluid (CSF). Polymerase Chain Reaction (PCR) may be used to differentiate wild strains from vaccine-like strains [5]. There are no approved antiviral treatments for polio, and the only possible prevention is through vaccination. Immunity is established by the administration of two types of vaccines i.e., Inactivated Polio Vaccine (IPV) and live-attenuated Oral Polio Vaccine (OPV). The Oral Polio Vaccine is more feasible to use as no professional health workers are required to administer it, and it is more cost-efficient. Hence, the OPV is used in mass polio vaccination campaigns in third-world countries including Pakistan. However, OPV has been known to cause Vaccine Associated Paralytic Poliovirus (VAPP), with the type 2 serotype being the most common strain of VAPP. The type 2 strain has now been removed from the Oral Polio Vaccine to limit the incidence of VAPP [6].

As polio is on the verge of eradication all over the world, there are still some countries that are facing hindrances in eliminating this virus among their peoples. Recently, there has been a hike in the number of cases of Polio Virus in Pakistan. The failure of government policies and planning when it comes to eradicating polio is evident form the fact that eleven cases of polio have been reported in the Northwestern region of Waziristan in 2022 [7]. Before this recent surge in cases, the last case in Pakistan was reported in 2021 when a child was diagnosed with the case of paralytic poliovirus. Reports suggest that the main causes of the hike in polio cases are false markings, bribery, and refusal of the general public to get their children vaccinated [8].

In Pakistan, for check and balance, the polio healthcare volunteers mark the thumb or finger of every child they vaccinate to ensure that he/she has received the vaccine dose. However, due to manipulation by certain tribals, unvaccinated children are falsely marked ensuring the healthcare setup in Pakistan that they had achieved their annual locums to vaccinate every single child. The recent rise in polio cases has unveiled this inhumane behavior. Some vaccine fanatics have also falsely reported the vaccination of their unvaccinated children, letting the government believe that their campaign is going smoothly and Pakistan is going towards polio eradication [8].

Pakistan has faced numerous challenges in its fight against polio. Lack of proper governance, geopolitical instability, insecurity, extremism, hindered access to remote areas, and most importantly the numerous misconceptions of the general public regarding the polio vaccine are just some of the reasons why Pakistan has failed to eradicate polio [9]. Certain vaccination myths have been quite common in Pakistan. For example, there was a time when there was a certain belief that this vaccination may sterilize the children and that children may lose their fertility. Furthermore, myths like Western countries installing microchips in the form of polio vaccination are still common. There have been numerous incidents in which teams of polio workers have been killed due to people's misconceptions about the polio vaccine. Just recently there has been an attack on a polio vaccination team in northwestern Pakistan in which one health worker and two policemen have been killed [10]. It is therefore essential that the health services of Pakistan take proper measures not just to ensure the safety of polio workers, but also to spread awareness among the people regarding the importance of vaccination.

Nationwide polio eradication campaigns have been occurring in Pakistan for the last 25 years. Owing to the recent hike in polio cases, Pakistan has also started its anti-polio campaign aiming to vaccinate 12.6 million children in 2022 [11]. The main purpose of these campaigns is not only to remove the misconceptions regarding the polio vaccine but also to initiate a polio-free environment all over Pakistan. Misconceptions can be dealt with by regular publication of problems that come up if parents refuse to get their child vaccinated. This sort of publication can be done through various social media platforms. Moreover, polio awareness seminars should be conducted frequently all over Pakistan, especially in tribal areas. The data of vaccinated individuals should be regularly checked to ensure that vaccination campaigns meet their annual vaccination goals. Furthermore, myths regarding vaccination should be regularly addressed by the local heads of tribal areas in collaboration with the Government of Pakistan.

## Provenance and peer review

Not commissioned, externally peer reviewed.

## Consent

Not required.

## Registration of research studies

1. Name of the registry: Not applicable.
2. Unique Identifying number or registration ID: Not applicable.
3. Hyperlink to your specific registration (must be publicly accessible and will be checked): Not applicable.

## Guarantor

Haroon Shabbir, Department of Medicine, Rawalpindi Medical University, Rawalpindi, Pakistan.

Sajeel Saeed, Department of Medicine, Rawalpindi Medical University, Rawalpindi, Pakistan.

## Ethical approval

Not applicable.

## Sources of funding

No funding required for the study.

## Author contribution

Haroon Shabbir: Study conception, write-up, critical review and approval of the final version. Sajeel Saeed: Study conception, write-up, critical review and approval of the final version. Muhammad Farhan: Write-up, critical review and approval of the final version. Khawar Abbas: Write-up, critical review and approval of the final version. Mohammad Ebad ur Rehman: Write-up and approval of the final version. Fahad Gul: Write-up and approval of the final version. Jawad Basit: Write-up and approval of the final version.

## Declaration of competing interest

All authors declared no conflict of interest.
